# Clinical analysis of lupus miliaris disseminatus faciei: a cross-sectional study and literature review

**DOI:** 10.3389/fmed.2024.1382526

**Published:** 2024-06-14

**Authors:** Yaqi Wang, Jiahui Li, Shuang Wang

**Affiliations:** Department of Dermatology, Second Affiliated Hospital of Xi’an Jiaotong University, Xi’an, China

**Keywords:** lupus miliaris disseminates faciei, clinical analysis, histopathology, image feature, dermoscopy

## Abstract

**Background:**

The clinical similarity of lupus miliaris disseminatus faciei (LMDF) and other papular granulomatous facial disorders often makes its correct diagnosis challenging. Diagnosis often requires the assistance of pathological examination, and dermoscopy can be used as an auxiliary and non-invasive examination method, however, the current findings remain incomplete.

**Objectives:**

This study aimed to summarize the clinical, histopathological and dermoscopic features of LMDF in the Chinese Han population and aiming to provide practical significance to correct diagnosis.

**Methods:**

109 patients of LMDF were collected in the Department of Dermatology, the Second Affiliated Hospital of Xi’an Jiaotong University from August 2015 to August 2023. The clinical and histopathological manifestations of all patients, as well as the dermoscopic image features of 44 cases, including background, follicular findings, vessels, and other structures, were summarized and evaluated.

**Results:**

The most significant histopathological features of LMDF in 109 cases is epithelioid granulomatous infiltrate in the superficial dermis, with or without caseation. The most significant dermoscopic features of LMDF in all 44 cases were orange structureless background (30/44), follicular plug (32/44), follicular white scar-like area (32/44), unspecific linear vessels (24/44), linear vessels with branch (24/44) and white streaks (18/44).

**Conclusion:**

Histopathologically, LMDF is characterized by the presence of epithelioid granulomatous infiltrate in the superficial dermis, with or without caseation. Dermoscopically, it exhibits a distinctive orange structureless background, follicular plug, follicular white scar-like area, nonspecific linear vessels, linear vessels with branches, and white streaks.

## 1 Introduction

Lupus miliaris disseminatus faciei (LMDF) is an uncommon chronic inflammatory and granulomatous disease, that commonly affects the central face (1). LMDF is characterized by multiple skin-colored to erythematous fleshy papules and nodules, typically affecting the cheek, periocular and perioral regions, especially the eyelids and earlobes (2). The lesions could resolve spontaneously in several years, may leaving varioliform depressed or atrophic scars (3). The histopathological features of LMDF are superficial dermal epithelioid granuloma with central caseation and follicular plugs. In addition, late stage lesions manifests as extensive perifollicular fibrosis (4).

The clinical similarity of LMDF and other facial inflammatory or granulomatous papules disorders brings great difficulties and challenges for dermatologists to correctly differentiate it (5). The levee shaped rash below the eyelids is suggestive. However, it is still easy to confuse with diseases such as acne vulgaris, granulomatous rosacea, granulomatous, and sebaceous gland hyperplasia (6, 7). The diagnosis of this disease relies on histopathological examination, but the pathological sampling is limited and invasive. In addition, the lesions often occur in the face, making it difficult for patients to undergo invasive pathological biopsy, which limits its application to a certain extent. This poses challenges for clinical doctors to make clear diagnoses and receive timely treatment.

Dermoscopy is a commonly used non-invasive examination in dermatology, which is simple and fast to operate and can clearly show the skin structure (8). Although it has been initially and mostly used for the auxiliary diagnosis of pigmented skin diseases, in recent years, the application of dermoscopy in the auxiliary diagnosis of infectious and inflammatory skin diseases has largely valued (9). This study retrospectively analyzed 109 patients with LMDF collected by our department in the past eight years, dermoscopic features were also evaluated among 44 patients with dermoscopy to explore the feasibility of dermoscopy as a non-invasive auxiliary diagnosis for LMDF.

## 2 Methods

This was a retrospective study of 109 patients with LMDF collected from the Department of Dermatology, the Second Affiliated Hospital of Xi’an Jiaotong University from August 2015 to August 2023. All enrolled patients were included in this study after being diagnosed by two associate chief physicians from the undergraduate department based on the patient’s clinical and histopathological findings. Among the 109 patients, 44 cases LMDF underwent dermoscopic examination and were conducted dermoscopic evaluation.

Dermoscope (DELTA20, German) and image acquisition equipment (CANONEOS600D, Japan) were used to take dermoscopic images at 10-fold magnification. All dermoscopic images were collected before treatment and all cases were histopathologically diagnosed by at least two senior pathologists.

The statistical analysis was conducted using Statistical Product and Service Solutions version 21.0 (IBM Corp., United States). Continuous data are presented as means (M) ± standard deviations (SD), while categorical data are expressed as numbers (*n*) and percentages (%). Comparison of categorical variables involved the use of the χ^2^ test, with Fisher exact test and continuity correction applied when appropriate. A two-sided *p* value <0.05 was considered statistically significant for the χ^2^ and Fisher exact tests.

## 3 Results

### 3.1 Baseline characteristic

In total, 109 patients (65 women and 44 men) of LMDF were included in this observation. The mean age of the study population was 38.3 ± 12.1 years (age range 8 ∼ 70 years). All cases involve the eyelids, with 107 cases affecting both eyelids bilaterally, while two cases specifically affect a single eyelid. Only three cases had skin lesions exclusively around the eyes, while the remaining patients had involvement in other facial areas. The most commonly affected areas, in descending order, were the nasolabial folds, lower jaw, forehead, cheeks, and upper jaw. The medical history varies from 1.0 to 120.0 months, with average duration of 14.8 ± 30.9 months. Among these 109 patients, 14 cases were mistakenly diagnosed as rosacea, common acne, hidradenoma, nodular sclerosis, sarcoidosis, and trichilemmal cyst.

**Figure 1:**
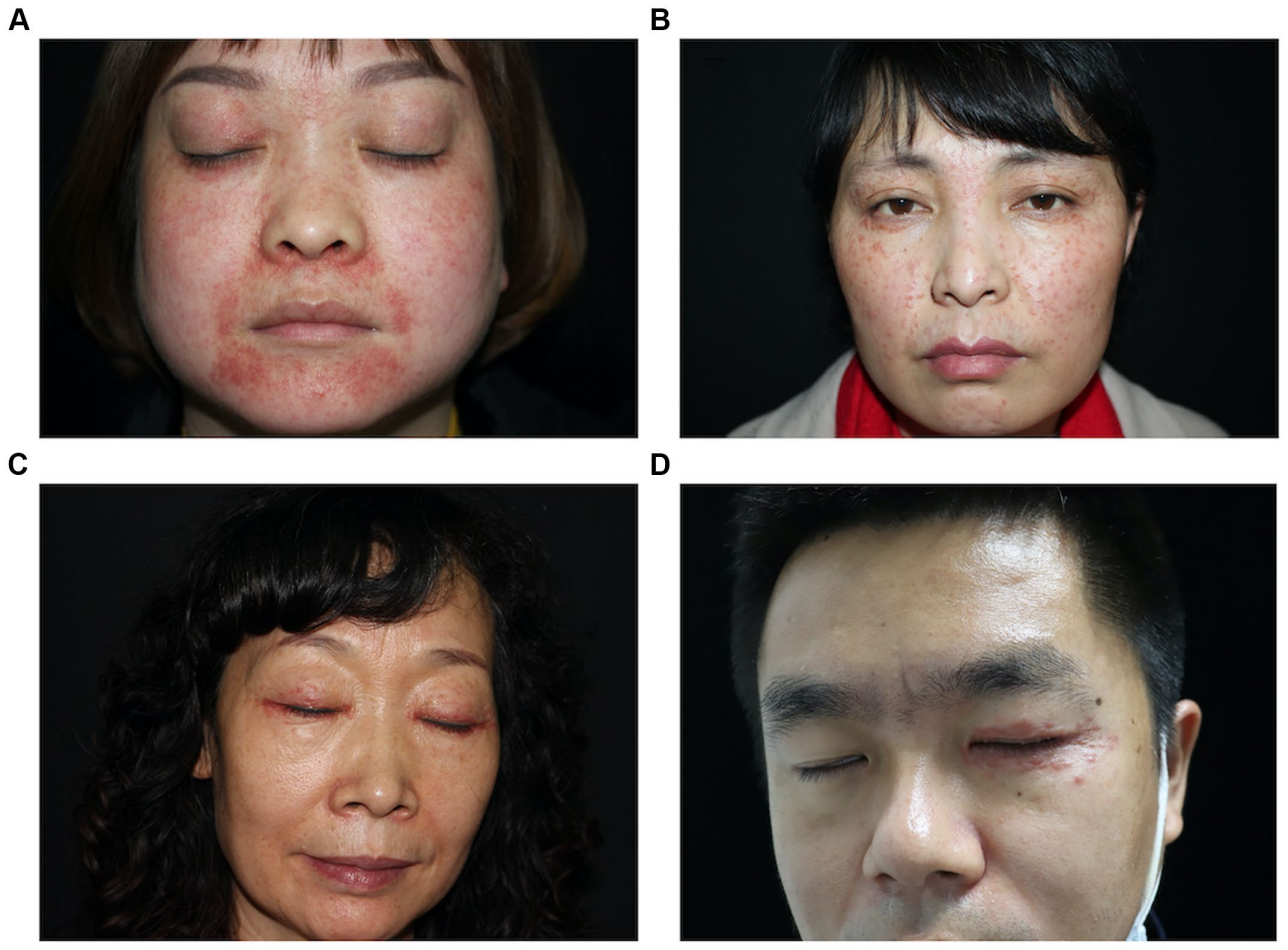
Typical clinical photos of patients with LMDF. **(A)** Erythematous papules on face. **(B)** Erythematous to skin-colored papules on face. **(C)** Erythematous papules over upper and lower eyelid. **(D)** Erythematous papules on left eyelid.

**Figure 2:**
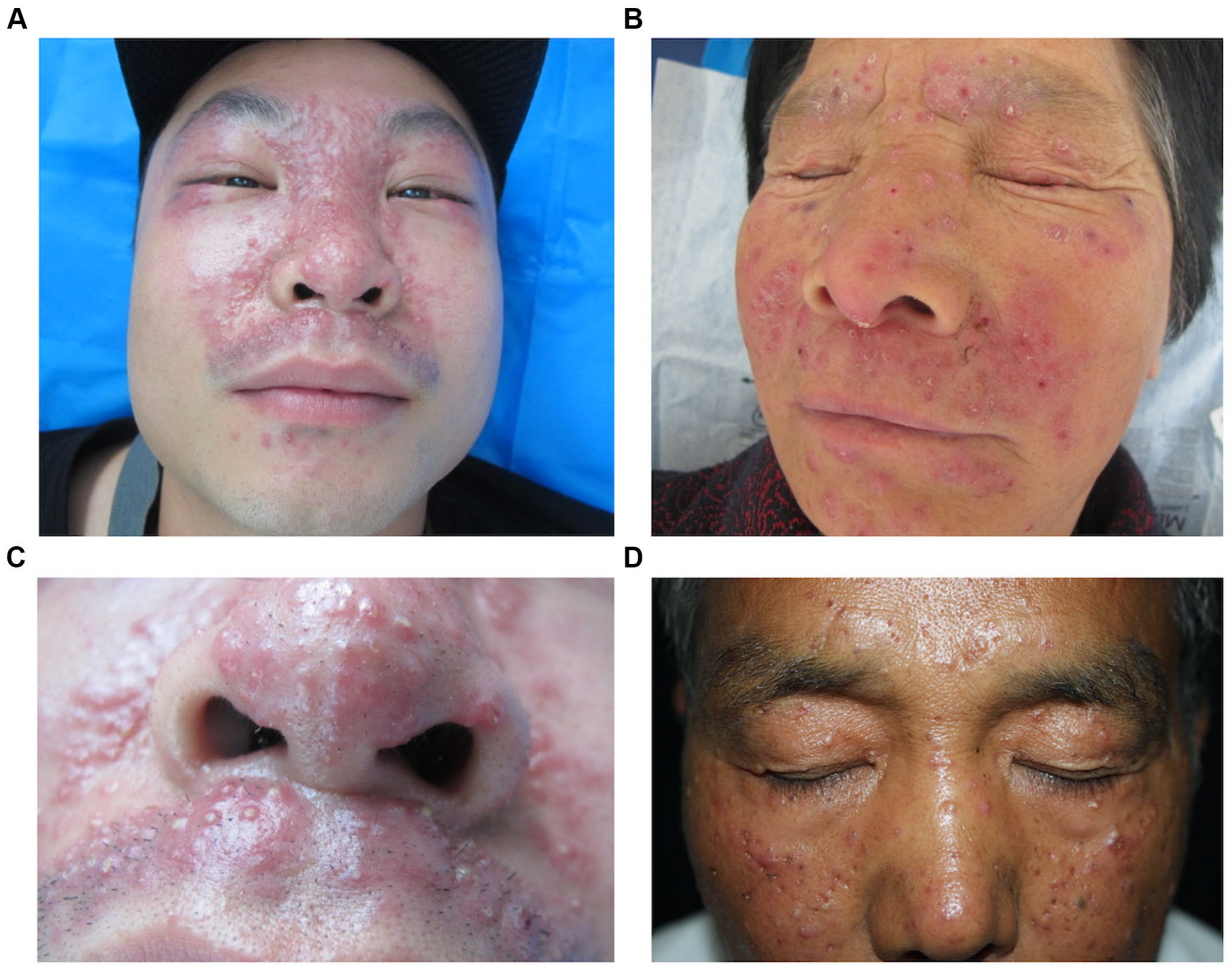
Various forms of LMDF lesions. **(A)** Erythematous papules and papules on face. **(B)** Scaly and crusted papular lesions. **(C)** Multiple pustules on the nose and upper lip. **(D)** Multiple atrophic erythematous scars over the forehead and cheek.

The skin lesions are mostly nodules ranging from millet seed to mung bean size. The majority of them exhibit infiltration, firmness, a slight elevation above the skin surface, and display a semi-spherical or slightly flat shape (Figures 1A–D). The lesions have a light red or reddish-brown color, featuring a smooth surface and a soft texture. The nodules appear intermittently, scattered and isolated, with some forming clusters of undetermined quantity. Some cases exhibit the formation of pustules (Figures 2A,B). Generally, there are no noticeable symptoms, but mild itching may accompany the condition (29.55%). Lesions in several patients ruptured upon scratching, revealing visible crusts on the surface. After healing, atrophic scars are frequently left behind (88.63%) (Figures 2C,D).

### 3.2 Histopathological results

**Figure 3:**
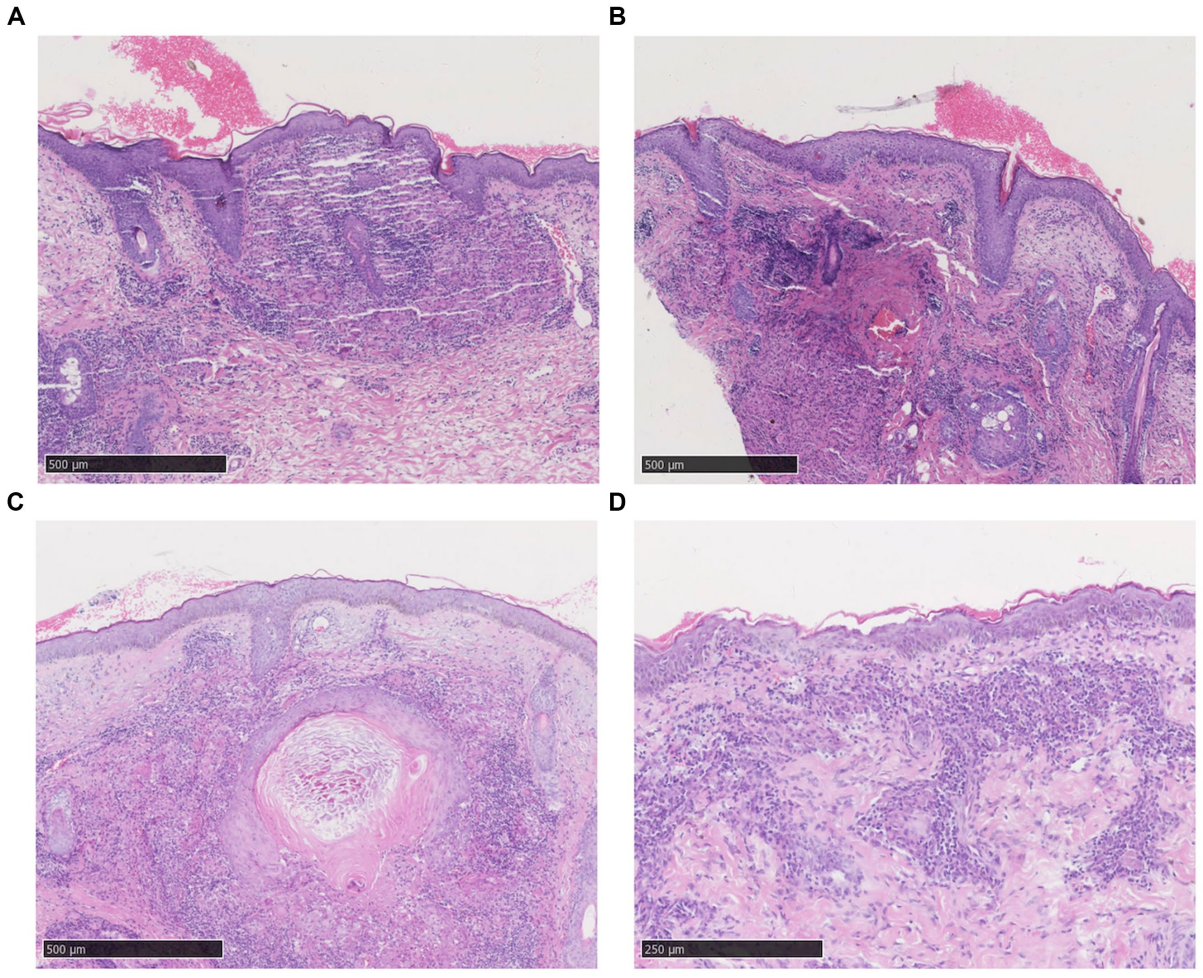
Histopathological features of LMDF. **(A)** Epidermal atrophy, scale formation, and superficial dermal epithelioid granuloma surrounded by dense lymphocyte infiltration. **(B)** Superficial dermal epithelioid cell granulomas with central caseous necrosis, surrounded by lymphocyte infiltration. **(C)** Dermal keratin cyst formation surrounded by lymphocytic infiltrates. **(D)** Higher magnification, the granuloma consists of epithelioid cells, lymphocytes and Langhans’giant cells with a central amorphous substance.

Among the selected 109 LMDF patients, light to moderate lymphocytic infiltration and scattered neutrophils were observed in the superficial dermis in all cases (Figure 3A). Based on the pathological manifestations, the cases were classified into the following types: 76 cases (69.72%) showed normal epidermis; 25 cases (22.94%) exhibited epidermal atrophy; 8 cases (7.34%) displayed epidermal hyperplasia; 73 cases (66.97%) showed simultaneous presence of epithelioid granulomas and caseous necrosis in the superficial dermis (Figure 3B); 73 cases (66.97%) had follicular plugs, and 4 case (3.67%) presented with a cornoid cyst (Figure 3C); 19 cases (17.43%) exhibited epithelioid granulomas without caseous necrosis (Figure 3D); 12 cases (11.01%) presented epithelioid granulomas along with abscess formation, accompanied by infiltration of neutrophils and eosinophils; and 5 cases (4.59%) showed only superficial dermal infiltration by mononuclear cells without other distinctive features.

### 3.3 Characteristic dermoscopic features of LMDF

**Figure 4:**
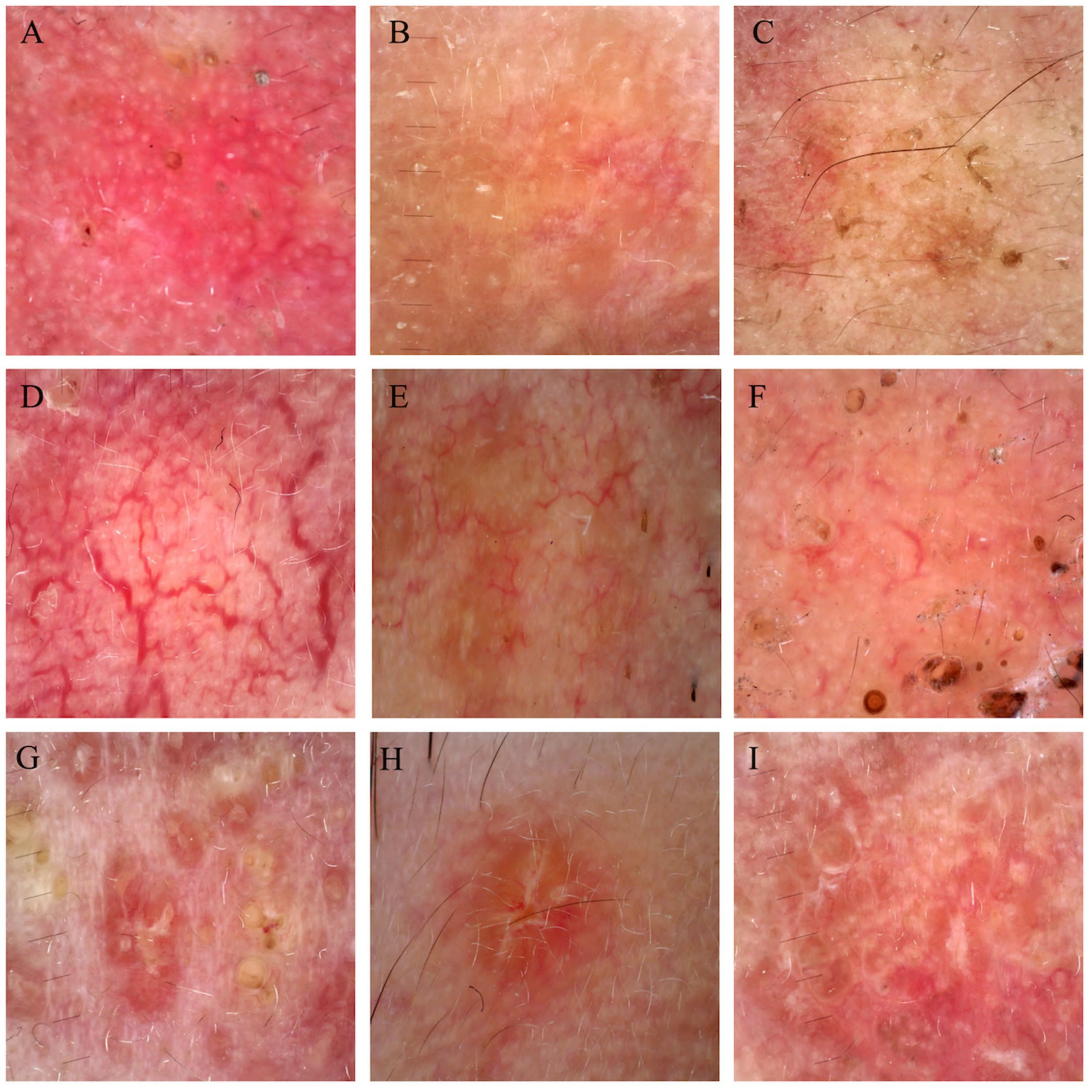
Dermoscopic figures of LMDF. **(A–C)** Background of LMDF in dermoscopy. **(A)** Red structureless areas; **(B)** orange structureless areas; **(C)** brown structureless areas. **(D–F)** Common vessels of LMDF in dermoscopy. **(D)** Reticular linear vessels characteristically arranged in polygonal networks; **(E)** linear vessels with branches; **(F)** unspecific linear vessels. **(G–I)** Follicular findings of LMDF in dermoscopy. **(G)** Follicular plugs; **(H)** follicular white scar-like area; **(I)** white steaks.

44 cases of LMDF were conducted dermoscopic evaluation, and almost 97.73% (43/44) of patients were diagnosed correctly with LMDF. Among these 44 cases of LMDF, 37 cases clinically diagnosed with LMDF, and 9 patients clinically misdiagnosed as rosacea, nodular sclerosis, hidradenoma or trichilemmal cyst. Dermoscopic images revealed orange, red or brown structureless background. Both follicular plugs and follicular white scar-like area were found in 32 (32/44) cases, and follicular red dots in 2 (2/44). For vessels, the most common pattern were unspecific linear vessels (24/44) and linear vessels with branch (24/44), followed by reticular linear vessels (8/44). White streaks were found in 18 (18/44) cases (Table 1; Figure 4). There were no significant differences observed in the dermoscopic manifestations between different age groups and genders.

**Table 1:** Dermoscopic features of LMDF.

Dermoscopic features	LMDF (*n* = 44)	Gender			Age		
		Male (*n* = 18)	Female (*n* = 26)	*p* value	≤40 (*n* = 26)	>40 (*n* = 18)	*p* value
Background							
Red structureless areas	10	4 (22.2)	6 (23.1)	>0.999	6 (23.1)	4 (22.2)	>0.999
Orange structureless areas	30	14 (76.9)	16 (61.5)	0.256	18 (69.2)	12 (66.6)	0.858
Brown structureless areas	4	0 (0)	4 (15.4)	0.226	2 (7.7)	2 (11.1)	>0.999
Follicular findings							
Follicular plug	32	12 (66.7)	20 (76.9)	0.684	18 (69.2)	14 (77.8)	0.778
Follicular red dots	2	2 (11.1)	0	0.316	2 (7.7)	0 (0)	0.64
Follicular white scar-like area vessels	32	14 (77.8)	18 (69.2)	0.778	20 (76.9)	12 (66.7)	0.684
Unspecific linear vessels	24	10 (55.6)	14 (53.8)	0.911	14 (53.8)	10 (55.6)	0.911
Linear vessels with branch	24	8 (44.4)	16 (61.5)	0.263	14 (53.8)	10 (55.6)	0.911
Reticular linear vessels	8	2 (11.1)	6 (23.1)	0.539	2 (7.7)	6 (33.3)	0.77
White steak	18	6 (33.3)	12 (46.2)	0.395	12 (46.2)	6 (33.3)	0.395

## 4 Discussion

LMDF skin lesions are primarily characterized by millet to mung bean-sized papules, with a smooth, semi-translucent surface, soft texture, and an apple jelly color under glass pressure. They are often distributed on the central and lateral aspects of the face, and ridge-like rashes beneath the lower eyelids are suggestive. Typically asymptomatic, these skin lesions usually spontaneously regress within 1–3 years, leaving atrophic and depressed scars after regression. Recurrence is generally uncommon after healing. This clinical condition is rare, and its diverse skin presentations can lead to misdiagnosis as common acne, hidradenoma, sebaceous adenoma, rosacea, and nodular sclerosis.

In this study, pathological examination of the 109 LMDF patients revealed mild to moderate lymphocytic infiltration and scattered neutrophils in the superficial dermis. Most patients exhibited epithelioid granulomas and caseous necrosis, with some showing neutrophil and eosinophil infiltration. Five cases had only mononuclear cell infiltration in the superficial dermis without other characteristic features. El Darouti and Zaher (10) investigated the histopathological manifestations of early, intermediate, and late-stage skin lesions in LMDF, finding that early lesions were primarily characterized by lymphocytic infiltration with minimal histiocytes and occasional nerve fiber cells. Intermediate lesions showed 20% involvement of caseous necrosis, while late-stage lesions were characterized by epidermal thinning and fibrosis. Our findings are generally consistent with previous reports.

In recent years, dermoscopy, as a non-invasive examination method, has become a crucial link between clinical and histological examinations (11). It serves as a key tool for evaluating pigmented and non-pigmented skin tumors (12). Additionally, it has gained recognition in fields beyond dermatology, such as vascular diseases, inflammatory conditions, infectious diseases, hair, and nail disorders (13, 14). Dermoscopy has emerged as an essential auxiliary diagnostic method in the diagnosis and differential diagnosis of LMDF. In this case series, 14 (14/109) cases were mistakenly diagnosed as rosacea, common acne, hidradenoma, nodular sclerosis, sarcoidosis, or trichilemmal cyst, resulting in delayed treatment and poor outcomes with residual atrophic scars. Therefore, a thorough understanding of the dermoscopic features of LMDF is crucial to inform clinical practices. Nevertheless, the dermoscopic characteristics of LMDF have not been fully explored. To further understand the efficacy of dermoscopy in LMDF diagnosis, we continued the study with the 44 patients who underwent dermoscopic examination. The dermoscopic findings of LMDF in 44 patients were described and compared in the present study, revealing that 7 cases were clinically misdiagnosed, but a correct diagnosis was achieved in 97.73% (43/44) of the cases by dermoscopy.

We observed some new dermoscopic features of LMDF beyond what has been previously reported. The orange structureless background with a follicular plug, considered a specific feature of LMDF, was the most notable dermoscopic finding in our study. Nevertheless, red diffuse structureless backgrounds are also frequently observed. Additionally, despite earlier reports indicating that nonspecific linear vessels are commonly seen in LMDF and lack diagnostic value, we observed that branched vessels and reticular linear vessels are also prevalent in LMDF. Besides, the dermoscopic manifestations, including the red structureless background and branched or reticular linear vessels, correspond to clinically observed bright red rash and, pathologically, to telangiectasia in LMDF.

While follicular plug holds diagnostic value, and most patients exhibit this characteristic, not all patients undergo this alteration, indicating that some cases might experience missed or delayed diagnosis (15). Follicular white scar-like area might histologically correspond to follicular fibrosis and white steaks might be related to dermis thickening and fibrosis (4, 16). The incidence of follicular scar-like area is as high as that of follicular plug; we observed that patients without follicular plug still presented with follicular white scar-like area. Therefore, follicular white scar-like area and white steak hold equivalent diagnostic value.

At the time of initial diagnosis, despite clinical misdiagnosis in 9 patients, dermoscopy revealed information not discernible to the naked eye. Given this circumstance, dermoscopy emerges as a crucial factor for dermatologists during clinical assessments (17). Serving as a distinctive auxiliary diagnostic method for LMDF, a dermoscope proves instrumental in raising suspicion of this diagnosis. Notably, this study contained the largest LMDF sample size to date, contributing significantly to the exiting body of knowledge on the subject.

## Data availability statement

The original contributions presented in the study are included in the article/supplementary material, further inquiries can be directed to the corresponding author.

## Ethics statement

The studies involving humans were approved by the ethics committee of Second Affiliated Hospital of Xi’an Jiaotong University. The studies were conducted in accordance with the local legislation and institutional requirements. Written informed consent for participation in this study was provided by the participants’ legal guardians/next of kin. Written informed consent was obtained from the individual(s), and minor(s)’ legal guardian/next of kin, for the publication of any potentially identifiable images or data included in this article.

## Author contributions

YW: Conceptualization, Data curation, Formal analysis, Investigation, Methodology, Project administration, Resources, Software, Supervision, Validation, Visualization, Writing – original draft, Writing – review & editing. JL: Conceptualization, Data curation, Formal analysis, Investigation, Methodology, Project administration, Resources, Software, Supervision, Validation, Visualization, Writing – original draft, Writing – review & editing. SW: Conceptualization, Data curation, Formal analysis, Funding acquisition, Investigation, Methodology, Project administration, Resources, Software, Supervision, Validation, Visualization, Writing – original draft, Writing – review & editing.
